# The Canadian Atlas of Child and Youth Injury: Mobilizing Injury Surveillance Data to Launch a National Knowledge Translation Tool

**DOI:** 10.3390/ijerph14090982

**Published:** 2017-08-30

**Authors:** Ian Pike, Jennifer Smith, Samar Al-Hajj, Pamela Fuselli, Alison Macpherson

**Affiliations:** 1 Department of Pediatrics, Faculty of Medicine, The University of British Columbia, Vancouver, BC V6H 3V4, Canada; ipike@bcchr.ca; 2 The BC Injury Research and Prevention Unit, BC Children’s Hospital, Vancouver, BC V6H 3V4, Canada; 3 Faculty of Health Sciences, American University of Beirut, Beirut 1107 2020, Lebanon; sh137@aub.edu.lb; 4 Parachute, Toronto, Ontario, ON M4P 1E8, Canada; pfuselli@parachutecanada.org; 5 School of Kinesiology & Health Science, Faculty of Health, York University, Toronto, ON M3J 1P3, Canada; alison3@yorku.ca

**Keywords:** children and youth, injury surveillance, knowledge translation

## Abstract

Child and youth injury prevention research in Canada has lagged behind other Organisation for Economic Co-operation and Development nations, despite existing surveillance systems and longitudinal data. A critical need to improve access to the available data, as well as need to tailor its display and interpretation, was identified by injury prevention stakeholders involved in research, policy, and practice. The Canadian Atlas of Child and Youth Injury Prevention (“the Atlas”) was developed to address this need. Following a series of iterative consultation meetings and a pilot testing session, the Atlas was scaled up with national data. Two testing sessions were held to evaluate the tools. The Atlas is comprised of three main components: data, indicators, and visualizations. The accessibility of the dashboard is enhanced by customization of data visualizations and data outputs to suit the user’s needs. Overall feedback indicated that the tools were easy to use, and that the interface was intuitive and visually appealing. The Canadian Atlas of Child and Youth Injury Prevention provides readily accessible information to injury prevention practitioners, policy makers and researchers, helping to chart pathways to success in improving the child and youth injury prevention system in Canada.

## 1. Introduction

Injury is the leading cause of death in Canada among those aged 1–44 years and claims the lives of more children <19 years of age than any other cause [1]. The economic burden of injury to children and youth in Canada is estimated to be approximately $5.8 billion per year [1]. Thus, the need for improved and enhanced injury surveillance and prevention initiatives is recognized as a priority at both national [2,3] and international levels [4]. Health Canada’s 2003 report on injury surveillance in Canada outlines the need for data that include the full spectrum of injury, from pre-event conditions and nature of injury, to course of treatment and rehabilitation [2]. Health Canada also emphasizes the importance of knowledge-user and stakeholder involvement in the conception and development of injury prevention products [2] as a medium for knowledge translation and mobilization research [3]. In British Columbia (BC), provincial and regional injury prevention decision makers recognize the need for prospective longitudinal data on the outcomes and consequences of injury, in order to support a business case for injury-related research and policy activities, and to assist prioritization [5]. Despite this awareness, Canada lags behind many other Organisation for Economic Co-operation and Development (OECD) countries in dedicated child and youth trauma and injury prevention strategy [3].

Injury prevention practitioners, policy makers, and researchers indicated that child and youth injury data in Canada was difficult to access, therefore they did not have what they needed to make decisions. Better access to data was needed—despite the existence of robust surveillance systems in Canada, with injury data available through data stewards such as Statistics Canada, the Drowning Prevention Research Centre, Canadian Injury Reporting and Prevention Program (CHIRPP), and the Canadian Institutes of Health Information (CIHI), among others [2]. Bringing these data sources together in a “dashboard” would permit information to be presented as temporal and spatial trends, and identify important issues not apparent with more traditional, paper-based approaches. In general terms, a “dashboard” is a graphical user interface that organizes and presents information in a format that is easy to read and interpret [6]. Dashboards allow users to quickly see the state of complex systems and are designed to assist in making informed and timely decisions for improvement [7]. In the public health arena, dashboards have been used for drug development [8], disaster preparedness [9], and employee health promotion and productivity [10]. This user-friendly web-based application seemed an appropriate vehicle to provide Canadian stakeholders with current injury indicator measures, and include the capacity to inform and monitor the development of injury prevention research and activity, policy and legislation, prevention programming, and the evaluation of new strategies.

The CIHR Team in Child and Youth Injury Prevention developed the Canadian Atlas of Child and Youth Injury Prevention (“the Atlas”; www.injuryevidence.ca) to answer this need, providing readily accessible information to injury prevention practitioners, policy makers, and researchers. The Atlas and dashboard is a unique resource and a first of its kind, serving as a model for other jurisdictions. The dashboard draws from multiple national data sources, provides a basis for decision-making and action, and to help chart pathways to success in improving the child and youth injury prevention system in Canada. Realizing the vision of the Atlas required the coordinated efforts of a multidisciplinary team over a period of six years; this paper serves to document that process and provide a comprehensive overview of the development, creation, testing, application, and dissemination of the final product, as well as evaluation from the standpoint of end-users.

### Background

The Atlas is comprised of three main components: data, indicators, and visualizations. Data tables are generated within the Interactive Data Online Tool (*i*DOT), indicators are defined within detailed text pages, and the visualizations are generated with Tableau software (Tableau Software; Seattle, WA, USA). The *i*DOT and dashboard both draw on the same datasets, but display the information in different formats, depending on the type of problem the user is attempting to solve. The accessibility of the dashboard is enhanced by customization of data visualizations and data outputs to suit the user’s needs. A series of iterative national consultation meetings were held to foster excellence in communication and encourage engagement through the development of a Canadian Child and Youth Injury Prevention Atlas and dashboard.

## 2. Materials and Methods

### 2.1. Consultation

Directly addressing a recommendation that Canada should choose a set of indicators comparable across institutions and organizations to monitor injury [3], the CIHR Team in Child and Youth Injury Prevention (the Team) defined and specified 34 injury indicators for Canadian children and youth, as well as a set of 27 indicators specific to First Nations and Inuit children and youth through a modified-Delphi process [11,12,13]. A meeting of national level data organizations (e.g., Stats Can, CIHI, etc.) was held in 2011 to determine availability and accessibility of injury data for the national indicators, with positive support to provide data to populate the dashboard.

Preliminary steps in the iterative development process undertaken by members of the team included: (1) assembling the operational requirements for the dashboard through input on what is meaningful to researchers, practitioners, and policy makers; and (2) populating the child and youth injury indicators with BC data as a proof of concept, before scaling up to the national level. This information was solicited through a series of presentations and discussions with key informant groups across Canada, and included feedback from over 100 potential dashboard users from research, policy, and practice organizations. Mock-up dashboard presentations and simulated working demonstrations were presented, coupled with directed questions regarding: the visual appeal and specific features of the dashboard; the utility of the indicator information; and the perceived functionality as a tool balancing utility, ease of use, and visual interest [2,6,7,8,9,10]. Specific questions related to the placement of “more important” information, grouping of indicators (e.g., easy comparison of mortality rate, potential years life lost, and cost), the “best way” to present certain types of data (e.g., charts, scorecards, gauges, and maps), and colour schemes.

The pilot interactive dashboard mock-up was built using Tableau software. The basic look and feel of the dashboard was incorporated into a working model using available BC hospital surveillance data (from the CHIRPP database) to understand the user experience and gather feedback. Advanced visualization techniques and interactive design principles were applied to design the Interactive Injury Dashboard according to pre-received user feedback based upon dashboard mock-ups. Multiple coordinated visualizations with embedded interaction capabilities, drill down and filtering techniques were integrated to help users easily retrieve injury data at multiple levels of abstractions. Throughout the project, the dashboard was discussed with the project team and modifications to specific visualizations were made to improve usefulness and understanding.

A meeting was held to demonstrate the pilot dashboard to eight identified injury prevention researchers and practitioners by having them interact with the dashboard as individuals and as part of a group. Participants were also asked to complete a questionnaire to solicit their feedback on the usefulness of the interactive injury dashboard and its potential to help stakeholders generate insights, build knowledge, and support their decision-making process. Methods and results of this testing session are reported elsewhere [14,15].

### 2.2. Data

Following the BC pilot demonstration, the Atlas was scaled up with national data. The datasets that are included in both the dashboard and *i*DOT included hospitalization and mortality data, drowning data, transportation data, and economic burden data [1]. These data were obtained from Statistics Canada, CIHI, the Drowning Prevention Research Centre, Parachute, as well as through individual provinces and territories and Transport Canada. Data sharing and confidentiality agreements were negotiated and approved prior to secure data transfers. As these agreements had to be negotiated individually, there were some delays in receiving the data. All data had to be coded in a numeric format to allow for programming on the *i*DOT. The dashboard visualizations on the other hand, were converted into text labels for programming into Tableau.

The mortality and hospitalization data required cleaning and preparation. This process included checking for missing data and information, coding external causes using the ICD 10 code breakdown, and creating categories by year, sex, age-group, cause, sub-cause, place of occurrence, type of injury, and body type injured. The hospitalization data included further data preparation for conversion by health region as well as coding by residential region and treating facility.

The drowning data was prepared by creating additional variables by region, safety device, and legal blood alcohol limits, as defined by the applicable provincial laws.

A more comprehensive approach was used for the transportation data, as each dataset received from the provinces was in a different format. Some data dictionaries matched while others were different. A consistent and standard data dictionary was developed based on common variables. Throughout the process, a highly qualified data coder with a diploma in health information management worked closely with the data team manager and programmer to optimize the coding system, ensuring accurate retrieval of data using other fields and free text in the database.

Each tool and visualization underwent a thorough testing process where data was verified using the original datasets. The tool was also sent to the data source organizations for testing, if they so requested. Different injury related scenarios were used to test the tool in its entirety.

### 2.3. Evaluation

A small group of key informant users tested the Atlas to provide feedback prior to “going live”. The key informants were injury prevention practitioners and policy makers representing national and provincial injury prevention organizations. Two consecutive testing periods during July and August 2015 involved seven participants each. Each user was individually oriented to the tool by a visual analytics expert, either in person or via a web-sharing platform, then they were presented with three scenarios to solve using the Atlas and given as much time as they needed to explore the tools (Box 1).

Box 1 **User Analysis Tasks.**

1. You are being asked to present at a meeting on the trends in Canada for suicide and homicide for 10–19 year olds from 2007–2010.
2. You are preparing a briefing note for the Ministry (or your municipality) regarding child pedestrian safety. What are your concerns? Be ready to provide evidence to support your stance.
3. A colleague has told you about a Potential Years of Life Lost visualization she saw on the Atlas/dashboard. How would you imagine using this in your work?
4. Take the next 5 min to explore the site. What is a typical thing that you would be asked to do as part of your daily work? Could this site help you with it?

On completion of the testing session, each user was interviewed. Users were asked to provide feedback on each component of the tool (dashboard, indicators, and visualizations), as well as their specific feedback about ease of navigation and architecture of the site, and overall experience using the Atlas. User feedback was grouped and examined across seven dimensions:

- Learnability—how easy is it for new users to perform the task?
- Intuitiveness—how obvious and easy is the task to accomplish?
- Efficiency—are users performing the tasks optimally? Are there ways to streamline and reduce the time it takes to complete the task?
- Preciseness—how prone to errors is the task?
- Fault tolerance—if a user makes a mistake while performing the task, how fast can he/she recover?
- Memorability—how easy is the task to repeat?
- Affordance—are interactive elements, such as buttons/links/input text boxes, related to the accomplishment of a task obviously interactive and within convenient reach?

Lastly, any specific problems relating to the structure and presentation of the site that were identified through this process were noted and corrected prior to going live.

### 2.4. Dissemination

Those same injury partners and stakeholders who were involved in the consultation and iterative design process offered to assist with further dissemination when the Atlas was ready to be launched. In addition, the Atlas was made known to the Canadian injury prevention community by sending out communications though existing list-serves, which reach approximately 500 members, as well as partner organization social media channels, and was presented at conferences, professional meetings, or via webinars, and posters at academic research days.

**Figure 1:**
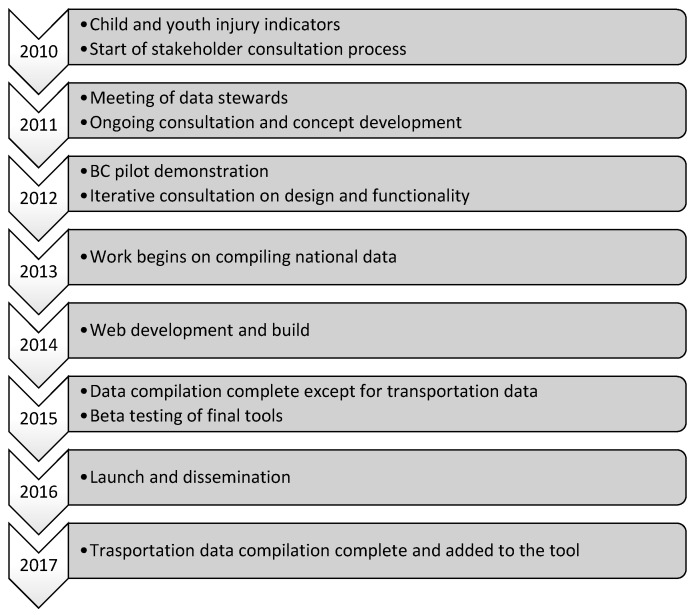
Atlas and dashboard timeline.

Google Analytics was used to track number of page views and user sessions, unique users and their locations, as well as length of time spent on the site, for a period of 12 months following the launch of the site on 17 March 2016. A timeline of the entire process is shown in Figure 1.

### 2.5. Ethics Statement

This project was reviewed and approved by the University of British Columbia Research Ethics Board (cert. # H09-01135). All testing session participants gave their written informed consent. To protect privacy, dashboard and *i*DOT data were amalgamated and values lower than five are not displayed.

## 3. Results

### 3.1. Product

#### 3.1.1. Dashboard

The dashboard includes seven sections for users to explore: injury deaths, hospitalizations, motor vehicle-related injury, sport-related hospitalizations, drowning, poisoning hospitalizations and economic burden. The dashboard incorporates intuitive visualizations including stacked bar chart, timeline trend, and geographic map. Multiple views of interactive representations serve to amplify stakeholders’ cognitive capability to understand and reason using dynamic injury data. 

The following elements were incorporated into the dashboard’s design and function:

1. Data overview and timeline trending visualizations for holistic understanding of the injury situation.
2. Drill down capabilities for additional levels of granularity.
3. Built-in interactive filtering to partition the data and focus on a specific interesting subset of the data.
4. Interactive distortion to show areas of interest with high level details while other areas are shown with low level details.
5. “Details on Demand” to interactively select parts of data to be viewed in more detail while providing an overview of the whole informational concept. “Details on Demand” is available by hovering the mouse over a specific area of the visualization.
6. “Interactive Linking and Brushing” to combine different visualization methods to overcome the shortcomings of a single technique. Brushing means to select a subset of injury data by clicking on it with the mouse to highlight the subset. This linking technique allows users to see how a particular data subset behaves in each of the visualizations in different windows of the dashboard.

**Figure 2:**
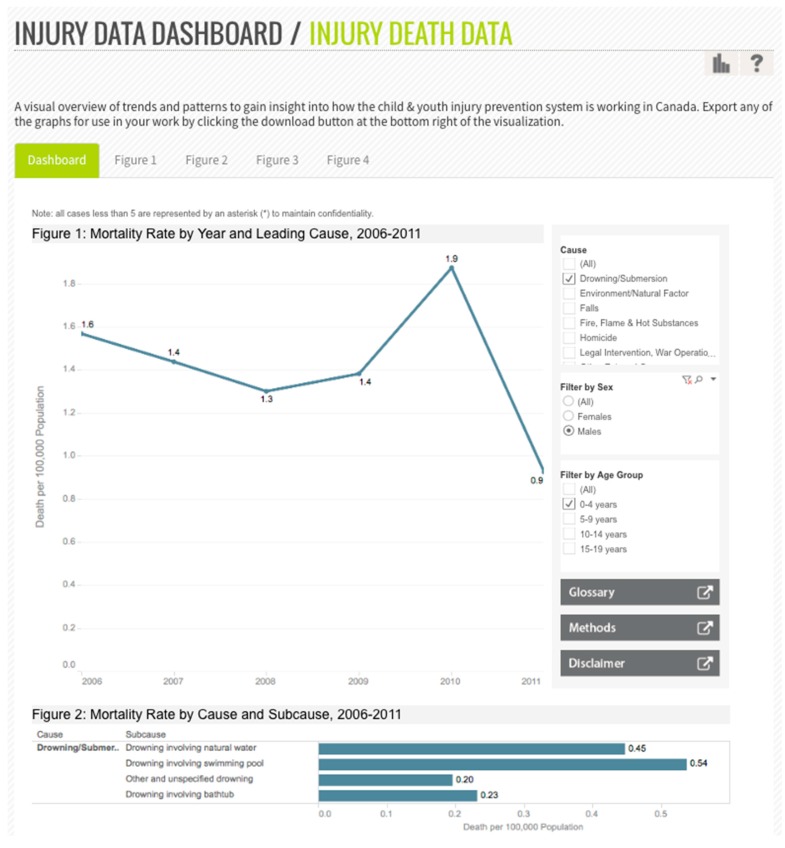
Dashboard output in graph display.

There are two main levels to the data presented in the dashboard visualizations with a further two sub-levels for some categories. The first level is an aggregated version where data are presented as a group. The second level is more detailed by breaking down the first level (Figure 2). Additional tabs allow stakeholders to explore the data using different displays and outputs, including regional maps and tables.

#### 3.1.2. *i*DOT

The main data selection page of the *i*DOT displays the data in six main sections, broken down further into topic areas. For example, the Injury Death Data section includes “region and year”, “demographics”, and “cause of injury”. Each topic area is further divided into sub-sections, which allow the user to select the information needed, such as unintentional drowning deaths in males, aged 0–4 years. The tool has been developed to allow easy navigation and minimal screen consumption. Therefore, all the parameters are stored under collapsible categories that expand when selected.

**Figure 3:**
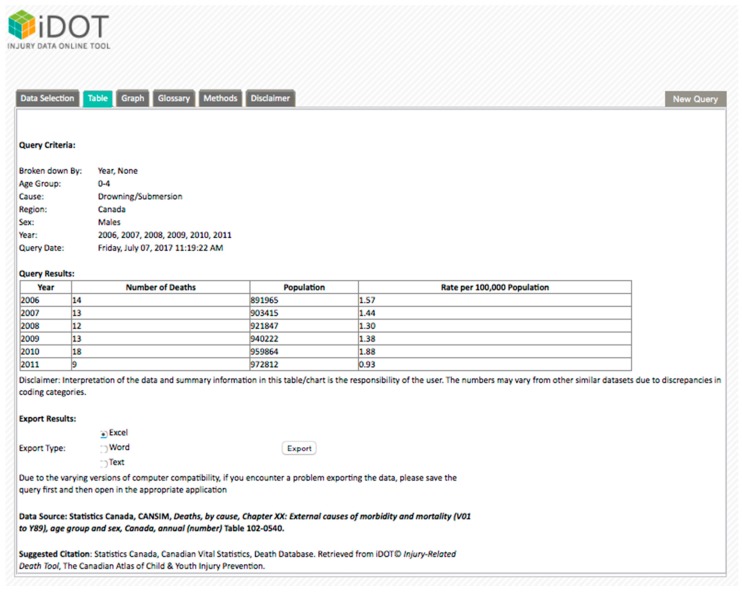
*i*DOT output in table display.

By selecting specific parameters in the search selection, outputs in the form of tables and charts are generated (Figure 3). A summary of selected parameters as well as the date and time of each search appears before each table or chart in order to allow cross-referencing of selections.

#### 3.1.3. Injury Research Insights

This section of the Atlas is content-based, and includes information about the indicators developed for the dashboard and *i*DOT tools by the CIHR Team in Child and Youth Injury Prevention [11,12,13], as well as application to real world scenarios and current research highlights. Each indicator is defined, along with key terms relating to the indicator, and the real-world importance of the indicator is illustrated in a lay language summary. Each insight provides easily accessible information to support interpretation of the data visualizations, thus facilitating knowledge translation for non-academic users.

### 3.2. Evaluation

Results of the final usability testing sessions are displayed in Table 1. Overall feedback indicated that the tools were easy to use, and that the interface was intuitive and visually appealing. Users were able to quickly learn how to use the tools and complete the tasks. Some technical issues were identified during the testing sessions, relating to placement of navigation features, as well as a need for troubleshooting support for certain data selection features to improve efficiency and precision. Users in the first testing session spent an average of 5 min and 26 s on each task, ranging from 0–15 min per task. The second testing session was not timed.

### 3.3. Dissemination

The Atlas was promoted via list-serve or newsletter to approximately 15 partner organizations, including Canadian Collaborating Centres for Injury Prevention, Institute for Work and Health, BC Injury Research and Prevention Unit, Parachute, Canadian Concussion Collaborative, and Ontario Health Promotion E-Bulletin and Knowledge Translation Canada, among others. The partners were in turn encouraged to disseminate the Atlas among their own list serves. The Atlas was presented at national and international conferences [16]. The Atlas was also promoted extensively on social media and is currently housed by Parachute.

In the 12 months following the launch of the Atlas on 17 March 2016, the website accumulated 8217 page views and 2983 sessions, with 1820 users spending an average of 3 min and 40 s, and viewing 2.75 pages per session. Approximately 60% of the website traffic was new users, and approximately 70% of all users were located in Canada. Users in Vancouver accounted for 18% of the Canadian traffic, with users in Toronto (9%), Ottawa (5%), Halifax (4%), Edmonton (2%), Calgary (2%), Kingston (2%), and Victoria (2%) comprising 8 of the top 10 cities.

## 4. Discussion

The Canadian Atlas of Child and Youth Injury Prevention is a unique, comprehensive resource, publicly and freely available for the use of injury prevention stakeholders, practitioners, policy- and decision-makers, and researchers across Canada. In answer to a call from the injury prevention community for better access to injury data, the Atlas is the first injury knowledge translation tool of its kind, with capabilities and features designed to promote decision-making and action. Involving data stewards from the very start of the consultation process secured their buy-in early on, paving the way for mobilizing the necessary data for testing pilot versions of the platform.

Extensive consultation with intended end-users of the final product was a key driver of the design and functionality of the tools. Users identified the need for customization, flexibility, and the ability to view select details of a subset of the data at the same time as the broader picture. The dashboard, *i*DOT, and injury insights were developed directly out of recommendations regarding the operational requirements and the child and youth injury indicators identified through the preliminary work of the team. Mock-ups built according to these foundational directives were then further developed and refined through an iterative consultation process with various injury stakeholders, which also served to enhance positive support for the Atlas within the injury prevention community.

Reflective of the fragmented nature of injury data in Canada, housed with various national, provincial, territorial or regional data stewards, the process of compiling, cleaning and linking the datasets for use in the dashboard and *i*DOT required a substantial investment of time and resources. As a knowledge-mobilization tool, the Atlas facilitates access to data by the injury prevention agencies and partners that may not have equivalent resources to compile the data to suit their individual needs. In this capacity, the Atlas may be considered as a first step in addressing inequitably resourced efforts to reduce child and youth injury in Canada.

Evaluation and testing of the Atlas prior to going live demonstrated the utility of the tool. Users reported very few technical errors or criticisms of the interface and content. Although potentially limited by small sample sizes, evaluation data reached saturation and was gathered from a wide range of injury prevention stakeholder types to increase confidence in the generalizability of the results.

Examination of the website analytics in the year following the launch of the Atlas revealed high engagement with the tool. Visitors to the site spent close to four minutes viewing multiple pages, suggesting that the content was of considerable interest to them and addressing the specific need for information that brought the users to the site in the first place.

Although the Atlas is still relatively new, updates to the data will become necessary to keep the tool relevant as time goes on. Securing the required funding to maintain and update the site presents an ongoing challenge, and data sharing agreements with data providers will need to be renewed as they expire.

To our knowledge, this is the first dashboard developed in partnership with the intended users, and the first time Canadian injury data has been summarized and available online in one place. However, there are some limitations. In some instances, data was not available from certain jurisdictions, most commonly the Territories and Quebec. Although a broad consultation was conducted nationally, it is possible that some stakeholders were not included. Finally, detailed data on mortality by province was not available, making comparison of death rates not possible. At the time of this writing, the Atlas is still new and dissemination efforts continue. As awareness of the Atlas continues to build, future evaluation of the Atlas will include assessment of impact on policy, practice, and research.

## 5. Conclusions

Child and youth injury prevention research in Canada has lagged behind other OECD nations, despite existing surveillance systems and longitudinal data. A critical need to improve access to the available data, as well as need to tailor its display and interpretation, was identified by injury prevention stakeholders involved in research, policy and practice. To answer this need, the Atlas was developed to provide a vehicle for enhanced knowledge translation and mobilization with the intended audience. The dashboard feature of the Atlas was designed to assist the user in making informed and timely decisions, and prompt action to improve child and youth injury prevention in Canada, by presenting user-identified indicator metrics in a visible and understandable fashion. Users who evaluated the Atlas prior to launch found very few technical errors and indicated that they found the Atlas highly functional, intuitive, informative, and visually appealing. Stakeholders involved in the consultation and development phases continue to be involved in dissemination of the end product following the official launch in 2016.

## Figures and Tables

**Table 1:** Qualitative results of the usability testing sessions

Dimension	*i*DOT	Dashboard	Insights
Learnability	Easy to learn and easy to go back and fix errors	Display can be visually overwhelming, but users were able to complete the tasks easily	Understandable information, definition of an “indicator” was still challenging for some
Intuitiveness	Very intuitive, as the format is similar to other form-based tools	Graphs and filters were easy to use and explore	The information was easily understood, although definition of “indicator” was still challenging for some
Efficiency	There is a period of learning how to use the tool—once this is achieved, use is simple	Information regarding how and when to use the tool is needed to support users and improve efficiency	This section is content based, so no difficulties with efficiency
Preciseness	Some confusion about how to achieve a breakdown using “cause” and “subcause” of injury	Some technical issues identified and resolved between testing sessions	Technical issue with PYLL indicator resolved prior to going live
Fault Tolerance	Easy to go back and fix errors	Some technical issues identified and resolved between testing sessions	Search function was useful
Memorability	Able to repeat a task easily	Able to repeat a task easily, although “reset” button needed to be relocated	Very easy to repeat the tasks
Affordance	Buttons to select “cause” or “subcause” of injury not immediately apparent	Once the user is familiar, the tool is easy to use	No issues with navigation identified
